# Is refractive error a factor affecting scoliosis?

**DOI:** 10.1371/journal.pone.0303324

**Published:** 2024-05-13

**Authors:** Jianru Cai, Yue Zhou, Xiaojuan Chen, Xiaobo Huang, Lele Li, Yan Zhu, Qi Cai, Jianping Huang, ZhiMin Sun

**Affiliations:** 1 Department of Ophthalmology, Second Affiliated Hospital of Nantong University, Nantong, Jiangsu Province, China; 2 Nantong Center for Disease Control and Prevention in Jiangsu Province, Jiangsu Province, China; Lorestan University, ISLAMIC REPUBLIC OF IRAN

## Abstract

**Background:**

Scoliosis is one of the most common surgical disorders of the pediatric spine. Refractive errors are commonly associated with vision impairment worldwide. However, it is currently unclear whether refractive error correlates directly with the development of scoliosis.

**Methods:**

A cross-sectional study was performed in 2023, and a stratified cluster sampling technique was employed among school-aged students in Nantong City, China. Univariate and multivariate logistic regression analyses were used to investigate specific correlations between scoliosis and related parameters; various types of refractive errors were also included in the study.

**Results:**

The prevalence of scoliosis among school-aged students was 2.2% in Nantong city. Multiple logistic regression analyses showed that myopia, hyperopia, astigmatism, and anisometropia were not correlated with the development of scoliosis (all, p≥0.05). Lower body mass index (BMI) [adjusted odds ratio (aOR) = 0.92; 95% confidence interval (CI): 0.88−0.95; p<0.001], living in rural areas (aOR = 1.40; 95% CI: 1.05−1.86; p = 0.020), and older age (aOR = 1.32; 95% CI: 1.25−1.38; p<0.001) had significantly higher risks of scoliosis.

**Conclusions:**

Refractive errors did not correlate with the development of scoliosis. However, BMI, living in rural areas and older age did correlate with the development of scoliosis.

## Introduction

Scoliosis is the most common spinal pathology in children and adolescents [1]. It is a growth-related disease that occurs during the rapid growth period of the musculoskeletal system before skeletal maturity. Scoliosis is associated with increased back pain, stress level, and a reduced health-related quality of life [2,3]. The progression of untreated idiopathic scoliosis may lead to severe deformities and may be accompanied by restrictive lung disease [4] and psychosocial issues [5]. In China, scoliosis has also been listed as one of the top three diseases endangering the health of adolescents [6].

Previous studies have suggested that several factors may contribute to the increased prevalence of scoliosis [7–9]. However, the identification of factors for positive screening for scoliosis has not been fully reported [7]. Refractive errors are the most common cause of vision impairment worldwide [10]. It has been confirmed that refractive errors are associated with poor reading and writing postures [11], and that poor reading and writing postures may accelerate the progression of scoliosis [12,13]. Refractive errors include myopia, hyperopia, astigmatism, and anisometropia [14]. Some researchers have found that patients with scoliosis also have myopia [15], but some studies also suggest that scoliosis and vision problems are considered to be unrelated [16]. In order to better control for confounding factors and study the relationship between refractive error and scoliosis, it is important to incorporate potential confounding factors in the literature [7,8] such as age, sex, BMI, and whether the subjects are urban or rural, in the same study.

The purpose of this study was to investigate whether refractive error was a factor affecting scoliosis, and to assess the prevalence of scoliosis in school-aged children in China.

## Methods

### Design and subjects

The Ethics Committee of the Second Affiliated Hospital of Nantong University in China approved this study (approval number: 2023KT123). All protocols used in this study followed the tenets of the Declaration of Helsinki [17]. All participants were informed of the content of this study. The parents or guardians of the participants, on behalf of the minor, agreed to participate in this study and signed an informed consent form, and obtained verbal consent from the minor participants. Adult participants voluntarily agreed to this study and signed an informed consent form. This school-based eye study was conducted from September 5 to September 20, 2023 in Nantong City. This is a moderately sized city of 7.744 million people living in the east coast of China.

A cross-sectional study performed in Chaozhou showed that the prevalence of scoliosis was 6.15% in 2018 [18]. A stratified cluster sampling method was used to sample urban and rural areas in Nantong City. To achieve a power of 80%, the sample size was calculated using the formula [19,20], n = t^2^pq/d^2^, assuming a design effect of 1.5 due to cluster sampling and a nonresponse percentage of 5% [*t* = 2 for a 95% confidence interval (CI), q = 1-p, d = 0.1 p]. The total sample size was at least 6,104. More samples were included in the protocol to ensure better multifactor analyses.

Participants were stratified by grade and age. Each class in each grade was selected through simple random sampling. All students in the selected classes were required to participate in this study. Using random sampling method, six schools in urban areas and five schools in rural areas were selected. The schools in the urban areas included two primary schools, two junior high schools, one regular high school, and two vocational high schools. Rural schools included two primary schools, two junior high schools, and one high school. In each school, at least two classes were randomly selected from each grade. At least 80 students were selected each time.

The inclusion criteria included all school-age children who could participate in the examination. If students suffered from other eye diseases such as glaucoma, cataracts, optic neuropathy, or eye injuries, they were not included in this study. Students who were unable to perform the Adam’s Forward bending test for any reasonable reason were not eligible to participate in this study.

### Scoliosis screening

Eleven medical workers formed a scoliosis screening team, including two senior physicians, two residents, and three nurses. Prior to commencing work, unified organization and training on screening methods were conducted.

The National Standardization Scheme for Scoliosis Screening (GB/T16133-2014) [21,22] was implemented as the screening standard for this study. The screening team was informed and trained by the two senior physicians about screening methods, posture examinations and use of the Bunnell scoliometer before the screening. The screening procedures, screening methods, posture examination, and the Bunnell Scollometer used before screening were all standardized. Screening methods included visual inspection of physical signs, Adam’s forward bending test (FBT), and measurement of the angle of trunk rotation (ATR) with a scoliometer [23]. A patient with a difference of 5° or more was considered to be preliminarily diagnosed with scoliosis. Separate examinations for male and female subjects were conducted. A slip or undershorts were required to be worn by participants for the examinations. Participants who tested positive for scoliosis were registered and informed, and they were recommended to go to a specialized hospital for further examination.

### Ocular examinations

Five optometrists and five ophthalmologists performed all eye examination procedures. All participants received standardized ophthalmic examinations. An automatic refractor (WSRMK-8000; Biobase, Shandong, China) was used to measure non-cycloplegic refraction. Each participant’s eyes were measured and recorded three times, starting with the right eye. Vision measurements were measured by a standard logarithmic liquid crystal tumbling E chart (WSVC-100; Qingdao Optometry, Berkeley, CA, USA) at 5 m, starting with the right eye. The average value of the three measurements was then analyzed. Further adjustments were according to the spherical, cylindrical, and axial results obtained by the automatic refractometer to obtain the best-corrected visual acuity (BCVA). For students suspected of having eye abnormalities, they were referred to a professional medical institution for further diagnosis and treatment.

Referring to the Refractive Error Study in Children surveys [24,25], spherical equivalent refraction (SE) was calculated as: SE = cylindrical degree×0.5+spherical degree. Myopia was defined as SE≤ -0.5 D. Emmetropia was defined as -0.5 D<SE≤0.5 D. Hyperopia was defined as an SE>+0.5 D. Cylindrical refractive error was expressed as positive cylinder form. Astigmatism was the absolute value of a cylinder ≥1.0 D. Anisometropia was defined as the SE difference of ≥1.0 D between eyes. Except for anisometropia, only the data collected from the right eye was used for analysis.

Basic information was recorded such as sex and age. Conventional physical examinations were conducted by well-trained health workers at local community health centers, including weight, height and blood pressure. The weight parameter was precise to 0.1 kilograms without wearing thick clothes. The height parameter was precise to 0.1 centimeters without wearing shoes. Body mass index (BMI) was calculated as weight (kg)/height (m)^2^.

### Statistical analyses

SPSS statistical software, Windows version 22 (SPSS, Chicago, Illinois, USA) was used for data processing. The characteristics of the research objects were represented as the mean±standard deviation, frequency, percentage, etc. According to the situation, the chi-square test or independent *t*-test was used to compare the differences between groups in scoliosis and refractive error parameters, as well as other related parameters. Univariate and multivariate logistic regression analyses were used to determine specific correlations between scoliosis and related parameters. In multivariate logistic regression analyses, all variables for multivariate logistic regression analysis were examined for multicollinearity, and the variance inflation factors for all variables were less than 5. The multivariable logistic regression model was established by forward stepwise selection. The Hosmer-Lemeshow test showed P>0.05 (x^2^ = 3.84, P = 0.871). The odds ratio (OR) and 95% CI for the associated factors were calculated, and in the multivariate logistic regression analyses were expressed as adjusted OR (aOR). A value of P<0.05 was considered statistically significant.

## Results

A total of 10,264 participants were invited to participate in the study. The completion percentage of participants out of school-aged children in Nantong City was 7.2%. A total of 9,607 participants were included in the statistical analysis, of which 5,127 (53.37%) were males and 4,480 (46.63%) were females. The response of participants was 93.60%. The mean of age was 13.40±3.54 years, ranging from 7−20 years. As illustrated in Table 1, the overall prevalence of scoliosis was 2.2%, the overall prevalence of myopia was 75.0%, the overall prevalence of hyperopia was 5.0%, the overall prevalence of astigmatism was 29.1%, and the overall prevalence of anisometropia was 25.7%.

**Table 1:** Prevalence of scoliosis and refractive error stratified by age.

Age	n	Prevalence of scoliosis	Prevalence of myopia	Prevalence of hyperopia	Prevalence of astigmatism	Prevalence of anisometropia
7−8	946(9.8%)	0.0%	30.3%	17.7%	16.3%	9.0%
9−10	1157(16.2%)	0.6%	49.8%	8.9%	16.3%	12.7%
11−12	1591(16.5%)	1.3%	73.2%	4.0%	25.9%	21.5%
13−14	1604(16.6%)	2.4%	84.5%	2.9%	28.6%	27.3%
15−16	1615(16.8%)	2.6%	91.8%	1.9%	34.9%	35.2%
17−18	1559(16.2%)	4.0%	93.0%	1.5%	40.9%	35.4%
19−20	735(7.6%)	4.9%	94.3%	1.0%	42.9%	39.2%
Total	9607(100.0%)	2.2%	75.0%	5.0%	29.1%	25.7%

Table 2 shows the distribution of basic demographic and ocular parameters in this study. In terms of the relationship between refractive error and scoliosis, scoliosis was more prevalent in the myopic group (2.58%) than in the non-myopic group (0.96%) (x^2^: 22.27, P<0.05). Scoliosis was more prevalent in the anisometropia group (3.08%) than in the non-anisometropia group (1.86%) (x^2^: 12.67, P<0.05). Hyperopia and astigmatism were not associated with scoliosis (all, p>0.05).In addition, Table 2 shows that scoliosis was related to age, systolic blood pressure, and diastolic blood pressure (all, p<0.05).

**Table 2:** Characteristics of the study participants aged 7−20 years old based on whether with scoliosis (ATR≥5°) or without scoliosis (ATR<5°).

Characteristic	Total (n = 9,607)	With Scoliosis (n = 209)	Without Scoliosis (n = 9,398)	p value
Sex, n (%)				0.730
Male	5127	114(2.22%)	5013(97.78%)	
Female	4480	95(2.12%)	4385(97.88%)	
Myopia, n (%)				0.000
Yes	7207	186(2.58%)	7021(97.42%)	
No	2400	23(0.96%)	2377(99.04%)	
Hyperopia, n (%)				0.277
Yes	477	7(1.47%)	470(98.53%)	
No	9130	202(2.21%)	8928(97.79%)	
Astigmatism, n (%)				0.060
Yes	2795	73(2.61%)	2722(97.39%)	
No	6812	136(2.00%)	6676(98.00%)	
Anisometropia, n (%)				0.000
Yes	2471	76(3.08%)	2395(96.92%)	
No	7136	133(1.86%)	7003(98.14%)	
Urban-rural differences, n (%)				0.56
Urban	5341	112(2.10%)	5229(97.90%)	
Rural	4266	97(2.27%)	4169(97.73%)	
Age (years)	13.40 ± 3.54	15.79 ± 2.76	13.34 ± 3.54	0.000
BMI	20.04 ± 4.58	20.20 ± 4.13	20.04 ± 5.59	0.611
Systolic blood pressure (mmHg)	112.48 ± 12.72	114.89 ± 13.08	112.43 ± 12.71	0.006
Diastolic blood pressure (mmHg)	63.24 ± 8.20	64.62 ± 7.47	63.06 ± 8.26	0.015

Table 3 lists the results of univariate and multiple logistic regression analyses. Using univariate logistic regression analysis, the results showed that participants of older age were more likely to suffer from scoliosis (OR: 1.24, 95% CI: 1.18−1.29, p<0.001). Compared with participants without myopia, participants with myopia were 2.74 times more likely to suffer from scoliosis (OR: 2.74, 95% CI: 1.77–4.23, p<0.001). Compared with participants without anisometropia, participants with anisometropia were 1.67 times more likely to suffer from scoliosis (OR: 1.67, 95% CI: 1.26–2.22, p<0.001). In addition, scoliosis was associated with participants with higher systolic blood pressure (OR: 1.05, 95% CI: 1.00–1.03, p = 0.006). and diastolic blood pressure (OR: 1.02, 95% CI: 1.00–1.04, p = 0.015).

**Table 3:** Univariate and multivariate logistic regression analysis of scoliosis among all participants (n = 9,607).

Characteristic	Univariate analysis			Multivariate analysis		
	Crude OR	95% CI	p value	Adjusted OR	95% CI	p value
Sex						
Male	Reference					
Female	0.95	0.72−1.26	0.73			
Myopia						
Yes	2.74	1.77−4.23	0.000			
No	Reference					
Hyperopia						
Yes	0.66	0.31−1.41	0.28			
No	Reference					
Astigmatism						
Yes	1.32	0.99−1.76	0.061			
No	Reference					
Anisometropia						
Yes	1.67	1.26−2.22	0.000			
No	Reference					
Urban-rural differences						
Urban	Reference	0.82−1.43	0.55	Reference		
Rural	1.08			1.40	1.05–1.86	0.020
Age	1.24	1.18−1.29	0.000	1.32	1.25–1.38	0.000
BMI	1.01	0.99−1.04	0.611	0.92	0.88–0.95	0.000
Systolic blood pressure	1.05	1.00−1.03	0.006			
Diastolic blood pressure	1.02	1.00−1.04	0.015			

OR = odds ratio; 95% CI = 95% confidence interval.

After adjustment for other characteristics, the results showed that various types of refractive errors were not associated with scoliosis (all, p>0.05). However, scoliosis was associated with participants with older age (aOR: 1.32, 95% CI: 1.25−1.38, p<0.001). Compared with participants in urban areas, participants in rural areas were 1.40 times more likely to suffer from scoliosis (aOR: 1.40, 95% CI: 1.05−1.86, p = 0.020). In addition, participants with low BMI were more likely to suffer from scoliosis (aOR: 0.92, 95% CI: 0.88−0.95, p<0.001).

## Discussion

The current research identified the prevalence and related factors of scoliosis incidence in Chinese school-age children, and focused on the relationships between different types of refractive error and scoliosis. The results showed that there was no correlation between various types of refractive error and scoliosis. The results also showed that the prevalence of scoliosis among school-age children in Nantong City, China was 2.2%. Factors such as higher age, lower BMI, and living in rural areas were associated with an increased probability of scoliosis.

Scoliosis has been listed as one of the top three diseases endangering the health of Chinese adolescents [6]. Screening for scoliosis in school-aged children is crucial for early detection, prevention of further deformities, and healthy child growth. The prevalence of scoliosis among school-aged children varies worldwide, with a percentage of 2.93% in Indonesia [26], 5.2% in Germany [27], 10.4% in Turkey [28], and in The Republic of Korea, the prevalence of scoliosis has even increased from 1.35% in 2002 to 6.17% in 2008, and is still rising [29]. In China, the government and education departments have not included scoliosis screening in routine physical examinations of school-age children [30]. There have been few studies carried out domestically, and there are significant differences in the prevalence of scoliosis in the Chinese population, with a percentage of 3.9% in Zhejiang Province [7] and 6.15% in Chaozhou [18]. In the current study, the prevalence of scoliosis among school-age children in Nantong City, China was 2.2%. Our study included all age groups of Chinese school-age children, and compared to previous studies, the prevalence may have varied due to the age of the participants. Age is an independent risk factor for scoliosis. In the population aged 7−8 years, there has been no positive screening for scoliosis. As age increased, the prevalence of scoliosis increased, which was similar to some previous results [7,31,32]. The prevalence of scoliosis was 4% in the population aged 17−18 years, and 4.9% in the population aged 19−20 years. Adolescence is a period of rapid development of scoliosis, as well as a critical period for recovery and correction [33]. Therefore, routine screening for scoliosis should be carried out in adolescents.

The relationship between BMI and scoliosis has been widely recognized. In the current study, although univariate logistic regression analysis showed that BMI was not associated with scoliosis (OR: 1.01, 95% CI: 0.99–1.04, p = 0.611), multivariate logistic regression analysis showed that BMI was a protective factor for scoliosis (aOR: 0.92, 95% CI: 0.88–0.95, p<0.001). The current research is consistent with previous findings, showing that participants with lower BMI are more likely to suffer from scoliosis [34–36]. Skeleton and muscle mass may be intrinsic driving factors for the occurrence and development of scoliosis, which is often accompanied by low bone density, abnormal bone quality, and atrophy and asymmetry of the paraspinal muscles [37,38]. Worthington et al. [39] suggested that malnutrition may play a crucial role in the etiology of scoliosis. BMI was a more comprehensive index of reflecting the body shape, but nutrition covers a wider range of fields. In addition to BMI, research still needs to be combined with other indicators. It is noteworthy that multiple logistic regression analyses showed that participants in rural areas were 1.4 times more likely to suffer from scoliosis than those in urban areas. This urban-rural difference may be due to the lower nutritional status of rural residents, when compared to urban residents, and to more serious health issues related to diet [40,41]. Therefore, it is necessary to direct more attention to implementing health education, referral, and follow-up intervention measures for children and adolescents who test positive for screening for scoliosis in rural areas.

Myopia is the main cause of visual impairment worldwide, affecting over half of Chinese adolescents [42,43]. As shown in Table 1, the prevalence of myopia, astigmatism, and anisometropia gradually increased with age (all, Ptrend<0.001). The prevalence of hyperopia tended to decrease with age (Ptrend<0.001). These results were similar to previous studies [44–46]. In particular, the prevalence of myopia increased from 30.3% in the age group of 7−8 years to 94.3% in the age group of 19−20 years, indicating the severe need for myopia prevention and control in Chinese school-age children. The change in refractive error with age is believed to be related to an increase in the burden on children’s eyes [42,47]. Scoliosis is also listed as one of the three major factors endangering the health of Chinese adolescents. Some studies suggest a potential link between scoliosis and vision problems, and previous studies confirmed that students with myopia were more likely to suffer from scoliosis [48]. However, there have only been studies on the correlation between myopia and scoliosis, and other types of refractive errors such as hyperopia, astigmatism, and anisometropia have not been reported. In 2018, Cai et al. [18] conducted a study on the incidence of scoliosis in Chaozhou, China, and multiple logistic regression analyses showed that myopia (OR = 1.49) was significantly associated with scoliosis. However, in their study, the diagnosis of myopia relied on survey questionnaires and the participants did not undergo refractive tests. In 2021, a study was conducted on possible factors influencing scoliosis in high schools in Yunnan Province, China, which reported that sex, age, and uncorrected visual acuity were important factors in the occurrence of scoliosis [49]. However, in their study, only sophomore high school students were included, and the age distribution was not provided. Therefore, there may be insufficient evidence to confirm that myopia is associated with scoliosis. Unlike previous studies [18,49], the current study included various types of refractive error. Among them, the prevalence of scoliosis was 2.58% in the myopic group, and 0.9% in the non-myopic group. Univariate analyses showed that myopia was associated with scoliosis. Participants with myopia were 2.74 times more likely to suffer from scoliosis (OR: 2.74, 95% CI: 1.77−4.23, p<0.001). However, after correction for factors such as age, BMI, and urban-rural differences, multiple regression analyses confirmed that various types of refractive error, including myopia, were not associated with scoliosis (all, p>0.05). Similarly, blood pressure factors also showed a correlation with scoliosis using univariate analysis, but did not show a correlation with scoliosis using multivariate regression analysis (p>0.05). We speculated that these results were caused by spurious or secondary associations between exposure and outcome.

There were some potential limitations in the present study. Scoliosis can be identified by forward bending tests, a scoliometer or both, and confirmed by radiology [50]. The lack of radiological diagnosis may result in low accuracy in detecting scoliosis curves [51]. Coelho et al. [52] found that using a referral criterion of 5° could identify 87% of patients with idiopathic scoliosis, and other studies have also suggested a good correlation between the measurement values of the scoliometer and radiographic analysis [53]. The use of screening results as the basis for preliminary diagnosis has also been supported by some research results [8]. Second, the study did not further classify scoliosis, and whether refractive error was an influencing factor for different types of scoliosis requires further research. Third, the diopters (D) used in this study were measured without ciliary muscle paralysis, which may lead to an overestimation of myopia prevalence [54]. In addition, the nature of cross-sectional research limited the determination of causal relationships. In the future, more potential predictors should be included in the study and continuous research should be conducted to collect more epidemiological data.

## Conclusions

Various types of refractive error were not associated with scoliosis, but lower BMI, age, and living in rural areas were associated with scoliosis. Case control studies, genetic studies, and long-term follow-up should be used in future studies to further clarify the mechanisms and relationships between scoliosis and other possible influencing factors.

## Supporting information

S1 File (XLSX)
